# Mobile App for Simplifying Life With Diabetes: Technical Description and Usability Study of GlucoMan

**DOI:** 10.2196/diabetes.8160

**Published:** 2018-02-26

**Authors:** Kaspar S Schmocker, Fabian S Zwahlen, Kerstin Denecke

**Affiliations:** 1 Institute for Medical Informatics Bern University of Applied Sciences Biel Switzerland

**Keywords:** diabetes management, patient empowerment, mobile health, self-care, chronic disease management, diabetes mellitus, mobile apps

## Abstract

**Background:**

Patients with diabetes can be affected by several comorbidities that require immediate action when occurring as they may otherwise cause fatal or consequential damage. For this reason, patients must closely monitor their metabolism and inject insulin when necessary. The documentation of glucose values and other relevant measurements is often still on paper in a diabetes diary.

**Objective:**

The goal of this work is to develop and implement a novel mobile health system for the secure collection of relevant data referring to a person’s metabolis and to digitize the diabetes diary to enable continuous monitoring for both patients and treating physicians. One specific subgoal is to enable data transmission of health parameters to secure data storage.

**Methods:**

The process of implementing the system consists of (1) requirements analysis with patients and physicians to identify patient needs and specify relevant functionalities, (2) design and development of the app and the data transmission, and (3) usability study.

**Results:**

We developed and implemented the mobile app GlucoMan to support data collection pertaining to a person’s metabolism. An automated transfer of measured values from a glucometer was implemented. Medication and nutrition data could be entered using product barcodes. Relevant background knowledge such as information on carbohydrates was collected from existing databases. The recorded data was transmitted using international interoperability standards to the MIDATA.coop storage platform. The usability study revealed some design issues that needs to be solved, but in principle, the study results show that the app is easy to use and provides useful features.

**Conclusions:**

Data collection on a patient’s metabolism can be supported with a multifunctional app such as GlucoMan. Besides monitoring, continuous data can be documented and made available to the treating physician. GlucoMan allows patients to monitor disease-relevant parameters and decide who accesses their health data. In this way, patients are empowered not only to manage diabetes but also manage their health data.

## Introduction

According to the International Diabetes Federation, approximately 642 million people worldwide will suffer from diabetes in 2040 [1]. Diabetes mellitus poses enormous challenges for patients and health carers. Once diabetes is diagnosed, lifelong self-management is critical for glycemic control with direct impact to long-term prognosis for the patients. Diabetes self-management includes self-monitoring of blood glucose, weight management, eating, and taking and managing medications. Furthermore, preventing and controlling diabetes complications (eye, foot, and renal) is important and requires regular checkups with physicians [2]. Care costs for chronic diseases are immense. Research showed that these costs can be reduced by supporting the self-management capabilities of patients [3]. Studies have proven that self-management allows patients to effectively deal with the challenges of chronic diseases and their treatment by reducing complications and symptoms, thus maintaining the level of quality of life [4]. With the rapid and ongoing growth of wireless connectivity and mobile phone availability, apps are increasingly considered interesting for supporting disease management. There is evidence from small studies that app use may have a beneficial effect on health outcomes [5]. The American Diabetes Association guidelines confirmed that apps may be a useful tool for monitoring diabetes and preventing complications [2]. With this in mind, we designed an app supporting diabetes self-management and digitized the existing paper-based diabetes diary.

Even though thousands of diabetes apps are available in the iTunes App Store and Google Play store for Android [6], these have limitations, which we address with our diabetes manager, GlucoMan. For example, the app mySugr allows a user to document blood sugar and other values uploaded from measurement systems [7]. DiaFit [8] supports uploading data from gadgets such as Apple Watch for fitness activity and glucose monitoring. Lithgow et al [9] claim that existing apps often do not support synchronization with a glucometer, although this feature is desired by patients. Existing apps support data export via email or sharing in social media platforms [10], but secure data export to a database that physicians can easily access through their information systems is not at all supported [10]. Arnhold et al [11] performed a systematic review on diabetes apps and found out that most of the 656 apps they reviewed provided only one function, such as documentation, information gathering, data forwarding, reminder, or therapy support. Further, they concluded that data transmission of health parameters to physicians is an important issue for future systems and is currently not well established.

The goal of this work is to develop and implement a novel mobile health system that digitizes the diabetes diary, enabling continuous monitoring of relevant data regarding a person’s metabolism, and addresses the limitations of existing systems. A multifunctional app was developed aimed at supporting patients with diabetes in managing their disease by enabling documentation, data communication, and information gathering. Data is stored on a health platform where it can be accessed by physicians and researchers after patient authorization.

## Methods

### Requirements Analysis

We developed GlucoMan within the context of the Hospital of the Future Live (Spital der Zukunft Live, or SDZL), a Swiss project involving 16 companies and 6 hospitals that focused on eHealth technologies to develop information technology (IT) solutions for future optimized health care processes [12]. The Institute for Medical Informatics of the Bern University of Applied Sciences executes SDZL on behalf of GS1 Switzerland to study to what extent IT can optimize public health sector processes such as information flow and logistics in a system that starts and ends at home and involves the patient, carers, family doctor, specialists, and the hospital and rehab clinic. The project SDZL runs from June 2016 to June 2018. Several partners from the project were involved in the development of GlucoMan through provision of technology (hospINDEX, MIDATA, see below) and assistance with the requirements analysis. This particular subproject ran from September 2016 to June 2017.

We developed the concept and collected requirements based on interviews and discussions with 3 doctors from the university hospital in Bern, 2 representatives from Diabetes Switzerland, the national diabetes association, and 2 patients with diabetes recruited from the authors’ personal environments. In this way, we assessed and considered the needs of health professionals and patients during the app development phase. Additional information was collected by reviewing scientific literature and teaching materials retrieved by searching PubMed using combinations of the keywords “mobile app,” “diabetes,” “diabetes management,” “patient empowerment,” “mobile health,” and “self-management.” The existing paper-based diabetes diary was used as a basis for app development and deciding on functionalities to be integrated.

### Knowledge and Technological Resources

Drug information was retrieved from hospINDEX (HCI Solutions AG) based on the Global Trade Item Number. hospINDEX contains article and partner data in XML format, and the referenced articles are linked to commercial and scientific data. The selection of the data covers around 220,000 articles. Additionally, hospINDEX provides clinical decision support data. The database contains information on allergies, interactions with food, use during pregnancy, maximum dosages, and more.

We used the open food databases openfoodfacts.org, openfood.ch, and fddb.info to import the values of carbohydrates of nutrition products. The current version of the prototype supports communication and data exchange with the MyGlucoHealth (Entra Health Systems) wireless Bluetooth glucose meter. We selected this device since it provides a wireless interface. Other devices such as FreeStyle Libre (Abbott Laboratories) have been assessed for integration but were excluded due to proprietary data formats or missing data transmission interfaces.

Data is stored on the MIDATA IT platform [13]. MIDATA.coop has developed an open source IT platform for the secure storage, management, and sharing of personal health data of any sort. The platform underwent 3 independent security checks before the first personal data were stored. MIDATA.coop has also established a clear trust-promoting governance framework. A developer's guide is available [14] that explains the general architecture of the platform and how apps and plugins can interact with it.

Our app is developed with the Ionic v2 Framework and Cordova (HTML, Cascading Style Sheets, Typescript); therefore, it can be built for multiple platforms (Android, iOS, Windows Phone, Blackberry, etc). The runnable version was only created for Android because the iPhone Bluetooth interface could not be used as required.

### Usability Study

The objective of the usability study is to identify usability problems and refine the design of the system to address the identified problems. Two subjects suffering from diabetes, 1 diagnosed with type 1 (male, age 57 years) and 1 with type 2 (male, age 60 years), were recruited from the author’s personal environment for the study. They also contributed to the requirements analysis. Diagnosed 12 years ago and 8 years ago, respectively, both test persons already have some years of experience with living with the disease. Additionally, 4 persons who are not diagnosed with diabetes were included in the study: 2 females, aged 73 and 38 years, and 2 males, aged 73 and 56 years, from the author’s broader environment (friends and relatives of colleagues who were not involved in the app development). All test persons use their mobile phones daily. None of them had medical training. For the usability test, they were asked to use the app installed on a separate device so the test conditions would be the same for all participants. They had no time in advance to get familiar with the app. Instead, they had to complete the tasks with only a brief verbal introduction to the functionalities by the study coordinator.

The usability test comprises 81 tasks concerning the different functionalities of the app. For example, the test persons had to navigate to the monitoring screen and add a new appointment or remove it. Another task required manually entering measurement values such as weight or pulse or importing data from the glucometer. From the nutrition screen, the test persons manually entered carbohydrate values for their meals or scanned products to import the carbohydrate data into the app.

The number of trials per task was recorded (ie, how often the task had to be started to finish it—immediately, second try, more than 2 trials). Additionally, the number of clicks needed to fulfill each task was collected. For each task, we assessed the optimal number of clicks beforehand and compared this number to the measured values. The test persons were asked to think aloud when problems occurred. After participants completed the tasks, overall feedback on usability of the app was assessed with an 8-question questionnaire.

Even though the number of participants in the study was low, previous studies from the human-computer interface literature found that 80% of usability problems can be found with only 5 research subjects [15,16]. Turner et al [15] even claims that the most serious usability problems can be revealed with only 3 subjects. The problem space determines the estimated required sample size [11]. The tasks to be supported by GlucoMan are well defined, and the problem space is limited compared to other software systems. Thus, the 6 persons included in our study are expected to be sufficient to determine the main problems related to usability of the app.

## Results

### Requirements

Based on the requirements engineering, literature reviews, and interviews, we identified the following features to be implemented in GlucoMan:

- Patient can access personal medical data related to metabolism independently from time and location.
- Defined members of a patient’s care team can view the measured values documented and shared by the patient regardless of time and location.
- Data such as glucose levels are transmitted automatically from measurement devices to the app.
- Patient can specify target rates as control measures for specific values and get immediate feedback on the last added measurement value relating to a defined target range.

### Architecture and System Functionalities

**Figure 1:**
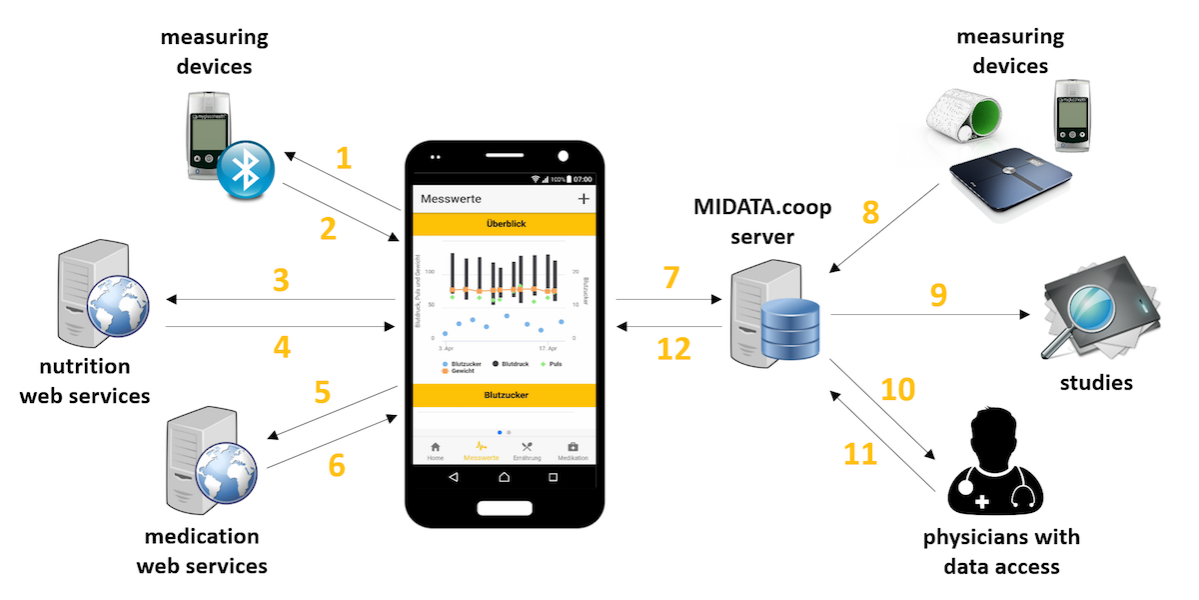
Concept and functionalities of the mobile app GlucoMan.

All collected data is made available to selected physicians and researchers when the patient provides access rights. The app connects to nutrition and medication Web services for carbohydrate and drug information (see section on technological resources). Data from medical devices are collected via Bluetooth. The concept underlying GlucoMan comprises a mobile phone app, data collection from medical devices or gadgets, and data storage (see Figure 1; the following numbers in the descriptions pertain to numbers in the figure).

To add new measurement values, Bluetooth-capable devices can be connected to the app (1). Upon data request, the actual measurements are imported to GlucoMan (2). In the current prototype, the glucose meter MyGlucoHealth is integrated as an example. Other devices can be added easily. Food names or data on nutrition (3) can be entered manually by the patient, and barcodes on food labels can be scanned. Current medication names (5) can be added manually or the drug barcode can be scanned by the patient. The scanned or entered products are searched in the drug or food databases, and relevant parameters are stored. For food items, information on carbohydrates (4) is retrieved, and for medications, article details such as product number and images (6) are retrieved. All collected and relevant data are uploaded to the personal account of the patient on the MIDATA platform (7).

The data exchange between GlucoMan and the platform MIDATA is realized using the Health Level Seven International (HL7) Fast Healthcare Interoperability Resources (FHIR) standard. This standard is based on resources, so-called observations, which are formatted in JavaScript Object Notation. Due the multiply encrypted data, MIDATA ensures a high safety standard, which is indispensable for personal medical data. The encryption of the key guarantees that the data are exclusively controlled by the owner. Without the key, the data can no longer be decrypted and are thus lost. Additional health data servers can be integrated easily as data sources via Bluetooth (8). From the MIDATA server, GlucoMan can retrieve relevant data for visualization (12). The data collected by the app and transferred to the MIDATA server can be made available by the patient for anonymized use in studies (9) or for monitoring purposes by the personal health care team (10). With the authorization of the patient, physicians are able to upload additional data like diagnoses, treatment, and personal notes to the account of a patient (11), which can be imported to GlucoMan where the patient can access it (12). GlucoMan shows all stored data aggregated in graph-like representations.

**Figure 2:**
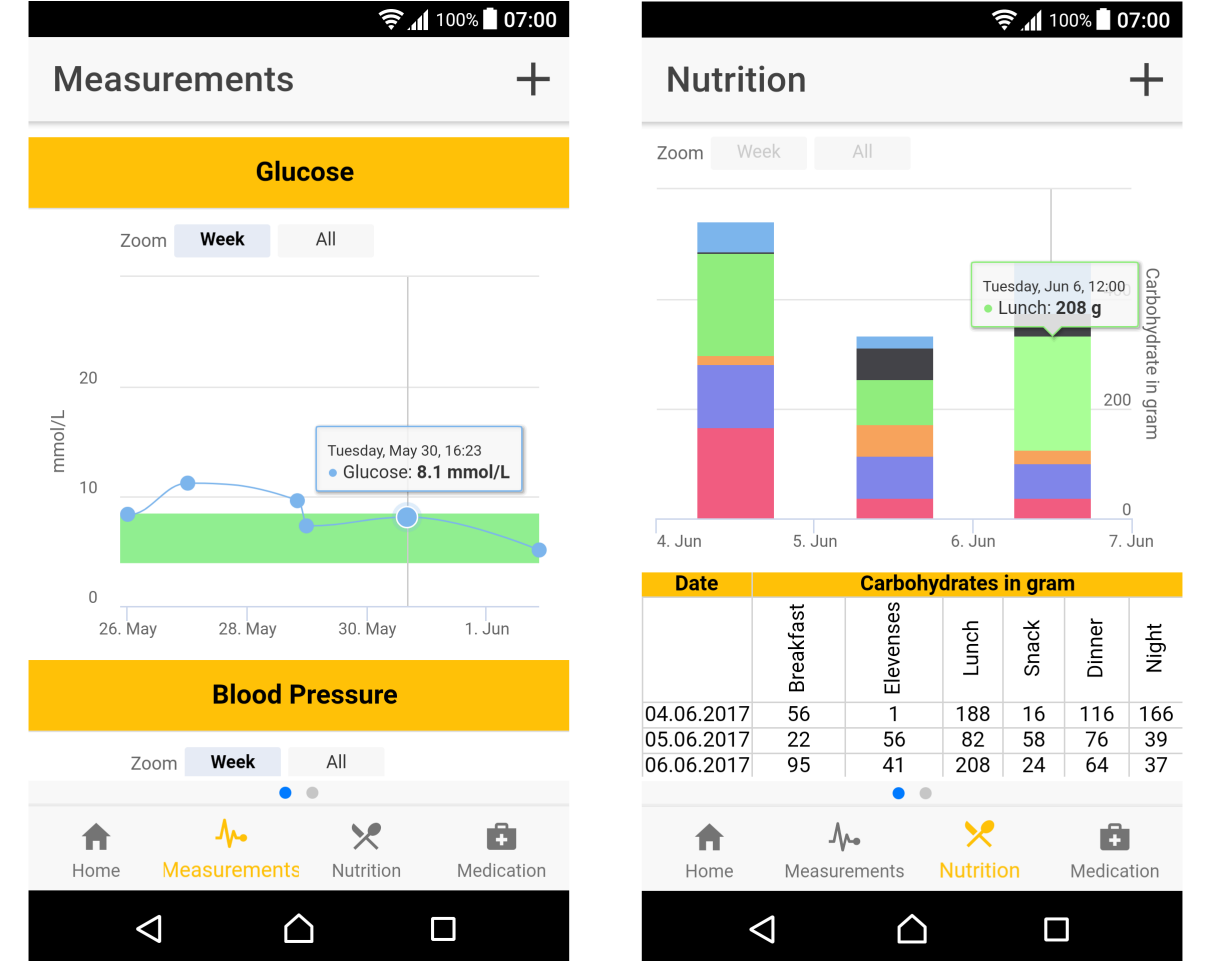
Left: GlucoMan screen showing glucose and blood pressure readings. Right: GlucoMan nutrition overview. Carbohydrates per meal are shown graphically and in a table for 3 continuous days.

The app allows specifying a measurement scheme comprising ranges for specific types of values and provides emergency support for persons who find a diabetic person in a situation of low blood sugar, advice for traveling with diabetes and on diabetes self-management, measurement values for HbA_1c_, blood pressure, and weight as measured in the monthly or bimonthly checkups. Different types of measured values such as blood sugar, blood pressure, pulse, and weight can be uploaded via Bluetooth (Figure 2). Information on nutrition and medication can be entered to enable monitoring (Figure 2). The app provides the functionality to import the carbohydrate value by scanning the bar code of a product to retrieve nutritional values ​​from food databases. If the database also contains the information on portion size, it is imported instead of the normal 100 g portion data. Before data are saved, the nutrition description, portion size (in grams), and carbohydrates (in grams) are displayed in an alert so that the user can still make changes. All measured values are visualized in an intuitive manner, enabling a user to monitor the changes in values. The different colors in the nutrition visualization represent the time of day. The size of the bar is determined by the number of carbohydrates (Figure 2). In order to record medication data, 4 types of medications are distinguished to enable a better overview: prescription medications, over-the-counter medications and supplements, insulin, and drug allergies. New drugs can be added by scanning the product bar code.

### Usability Study Outcomes

In general, the app has been rated as easy to use by the 6 subjects. The descriptions of buttons were understandable. Some interactive functionalities were not recognized: checkboxes that could be selected or that a further view could be obtained by swiping. Surprisingly, the 2 oldest study participants (aged 73 years) had no problems identifying the swiping to change the view.

Most of the features were instantly recognized and completed in the desired number of clicks. Five out of 6 test persons managed to complete the majority of tasks in 1 trial. One test person completed 8 tasks in 3 trials, 15 tasks in 2 trials, and the other tasks in 1 trial. For tasks related to adapting the visualizations or defining ranges for values, more than the expected number of clicks were made. The reason was that the desired functionality was captured in the Options menu which was hard to find. The test persons desired explanations on the possible options that could be adapted in the app (eg, changing the measurement schema) and mentioned that it would be helpful to get more obvious hints when pages can be swiped. The 2 oldest test persons requested a larger font size and larger checkboxes.

The 4 test persons who did not have diabetes were impressed with the possibilities of the app and in particular liked that the information was provided in a clear manner and no irrelevant data were presented or collected.

The 2 test persons with diabetes had different types and considered certain functions more or less relevant and evaluated them differently. In the case of type 1 diabetes, several blood glucose measurements have to be made per day. This requires monitoring the measured values ​​more often than with type 2 diabetes, where glucose is measured only 1 or 2 times daily.

The test person with type 2 diabetes stated that he would use the app in future if the diet in his therapy gains in importance. This person considers the data import by Bluetooth or barcode very practical.

The test person with type 1 diabetes currently measures his blood glucose level with a continuous measuring device. In order for the app to be used by him, a connection of his current measuring device needs to be enabled to import the values. In general, he considers the barcode detection a very helpful option. It would be beneficial if not only carbohydrates of ready-made meals could be calculated and recorded because this test person normally eats self-cooked food or in a restaurant.

## Discussion

### Principal Findings

The purpose of the proposed app GlucoMan is to relieve the patient from the duty of entering or manually documenting measured data for monitoring purposes and provide a user-friendly overview of available measurements. In this way, the patient is put into the position of managing and monitoring his or her diabetes—the patient is empowered to understand the disease. The data collection feature was confirmed to be useful by the person with type 1 diabetes; he currently has to record such values manually in the diabetes diary.

GlucoMan stores the data on the MIDATA IT platform. For further use of the stored data, it is possible to authorize other users of the platform to access the data or open it for use in studies. For example, researchers can use the data for finding hidden patterns in the diabetes data of a larger population. In this way, scientists can have access to anonymized data of a large population in the future. The treating physician with an account on MIDATA can inspect the recorded measurements and add treatment- and diagnosis-relevant data regardless of location and time.

By scanning the barcode of a food product or drug, the patient can obtain additional information on carbohydrates or medication doses. Data entry is simple and easy to use with the barcode scanner. Bluetooth-connected measurement devices transfer data directly to the system without any media break.

### Comparison With Prior Work

Functionalities of existing diabetes apps vary. Hood et al [17] distinguished apps that support monitoring tasks (diabetes-specific self-management tasks, weight and blood pressure tracking) from those that have educational purposes. Few apps provide personalized feedback or content. Most apps are equipped with glucose tracking, calorie counting, activity tracking, and education. Lithgow et al [9] found out that collecting data directly from a glucose meter is a feature missing [9] or supported only by a few apps [8]. Our app addresses this limitation.

Measurement devices normally support uploading values to the user’s account. Often, this data can neither be used from these platforms by external parties nor easily analyzed by a physician. Furthermore, existing diabetes management apps support data-sharing only in social networks or by export via email. By storing data on MIDATA, the patient remains the owner of his data and can provide access rights to selected physicians or even offer the data to clinical studies. This is in contrast to portals from hardware providers such as Whitings or Fitbit that store the data on proprietary platforms without giving any rights to the patient. Storing data on MIDATA can be recognized as a first step toward an electronic health record that integrates clinical and personal health data. All treating physicians can access the personal health data of a patient. This is a unique feature compared to existing apps. Additionally, no other app could be identified that uses HL7 FHIR for data transmission even though it is obvious that the use of standards is important for achieving interoperability and data reuse.

Whereas several Bluetooth and Internet of Things measuring devices already on the market for common vital signs like blood pressure, pulse, and weight give access on the measured data, many manufacturers of blood glucose meters are implementing proprietary protocols. For this reason, it is impossible for third-party systems to access or process the data. Our concept allows easily integrating data from gadgets or medical devices when accessible data protocols are provided. A future extension of the app would be the integration of an insulin pen such as inPen (Companion Medical) which would enable the person to also track insulin doses.

The open question for our app and also for many other available systems is the usefulness for patients in managing their diabetes. Studies by Hou et al [18] showed that mobile phone apps have the potential to improve glycemic control in the self-management of diabetes. However, they also concluded that younger patients were more likely to benefit from the app use. Additionally, a randomized controlled trial by Quinn et al [19] found that traditional intervention methods could not provide adequate blood glucose control, but a mobile diabetes intervention method improved clinical outcomes. The US Food and Drug Administration has approved some apps for diabetes management. This shows that apps can be useful in this context.

Jo et al [20] tested the app Healthy-note with patients and found out that effective support can be achieved when the app provides suggestions for lifestyle adaptations and helps in monitoring those. According to their results, interaction with the patient is important for setting goals and providing continuous encouragement. Through the specification of ranges for the values in GlucoMan, a patient is enabled to set goals for specific measurement values. Including features for encouraging a patient to work toward achieving the goals is still open for future work.

### Limitations

The user study involved 2 persons diagnosed with diabetes and 4 persons not diagnosed with diabetes. The latter could judge and test the functionalities but not the relevance of the app for diabetes management. However, we were able to identify main usability problems during the tests. Even though we involved the national diabetes association, it was not possible to recruit more patients in the short time available for this project. Even though the test persons were recruited from the personal environment of the authors, we are convinced that this does not affect the results of the usability study; the test persons completed defined tasks and number of clicks were counted, which is an objective measure. A comprehensive clinical study for testing the impact of GlucoMan on the self-management capacity and studying the usability of the system is currently planned.

Even though the concept enables data access by physicians, a corresponding user interface or data integration with existing information systems was not implemented as part of this work. This remains open for future work. We developed the connection to one specific glucose meter. To connect another medical device to the app, technical documentation needs to be available for the device that provides details on data requests and corresponding responses. For using the app in other countries than Switzerland, the underlying drug database would need to be changed. The hospINDEX only allows recognizing drugs that are approved on the Swiss market.

Currently, GlucoMan only enables entering data from products that have a barcode or where the patient enters the carbohydrates manually. A future extension is the integration of the GoCARB [21], a mobile system that allows taking a photo from a plate with food and calculates the carbohydrates automatically. This would clearly simplify the collection process for self-cooked meals.

### Conclusion

Collection of data on a patient’s metabolism can be supported with a multifunctional app such as GlucoMan. Besides monitoring, continuous data can be documented and made available to the treating physician. GlucoMan allows patients to monitor disease-relevant parameters and decide who accesses their health data. In this way, patients are empowered not only to manage diabetes but also to manage their health data.

## Abbreviations

FHIR: Fast Healthcare Interoperability Resources
HL7: Health Level Seven International
IT: information technology
SDZL: Spital der Zukunft Live (Hospital of the Future Live)
